# PDB-UF: database of predicted enzymatic functions for unannotated protein structures from structural genomics

**DOI:** 10.1186/1471-2105-7-53

**Published:** 2006-02-06

**Authors:** Marcin von Grotthuss, Dariusz Plewczynski, Krzysztof Ginalski, Leszek Rychlewski, Eugene I Shakhnovich

**Affiliations:** 1 Department of Chemistry and Chemical Biology, Harvard University, 12 Oxford Street, Cambridge, Massachusetts 02138, USA; 2 BioInfoBank Institute, ul. Limanowskiego 24A, 60-744 Poznan, Poland; 3 Bioinformatics Unit, Department of Physics, Adam Mickiewicz University, ul. Umultowska 85, 61 614 Poznan, Poland

## Abstract

**Background:**

The number of protein structures from structural genomics centers dramatically increases in the Protein Data Bank (PDB). Many of these structures are functionally unannotated because they have no sequence similarity to proteins of known function. However, it is possible to successfully infer function using only structural similarity.

**Results:**

Here we present the PDB-UF database, a web-accessible collection of predictions of enzymatic properties using structure-function relationship. The assignments were conducted for three-dimensional protein structures of unknown function that come from structural genomics initiatives. We show that 4 hypothetical proteins (with PDB accession codes: 1VH0, 1NS5, 1O6D, and 1TO0), for which standard BLAST tools such as PSI-BLAST or RPS-BLAST failed to assign any function, are probably methyltransferase enzymes.

**Conclusion:**

We suggest that the structure-based prediction of an EC number should be conducted having the different similarity score cutoff for different protein folds. Moreover, performing the annotation using two different algorithms can reduce the rate of false positive assignments. We believe, that the presented web-based repository will help to decrease the number of protein structures that have functions marked as "unknown" in the PDB file.

**Availability:**

and

## Background

Over 30 structural genomics centers have been established worldwide with the common goal of large-scale, high-throughput structure determination using X-ray crystallography and NMR[1]. One challenge is to predict the function of the proteins from their three-dimensional structures, primarily those which have no detectable sequence similarity to any protein of known function[2]. Currently, the total size of the Protein Data Bank (PDB)[3] is more than 32,000 entries, which contain over 29,000 different (63,000 redundant) protein chains. Many of the PDB chains have been mapped to Enzymatic Classification (EC) numbers *via *the Swiss-Prot database[4]. The mapping information has been presented as a PDBSprotEC database [5], which is available on the Internet. SCOPEC [6] is another web-based repository which is similar to PDBSprotEC collection. The SCOPEC set contains a description of the protein catalytic domains with assigned enzyme function. Prediction of protein function has been conducted using sequence similarity in both web-accessible databases. There is no doubt the PDBSprotEC and SCOPEC databases are full of very useful EC number assignments. However, none of these services contains predictions for proteins that have no sequence similarity to known enzymes. Moreover, neither PDBSprotEC nor SCOPEC includes any data for recently deposited PDB structures. The "youngest" annotated in PDBSprotEC or SCOPEC protein was released by PDB in August 2004 or in February 2003, respectively. Therefore, we decided to use the structure-function relationship [7-9] for automatic assignment of the EC number to 499 protein structures that came from the structural genomics centers and whose function is marked as "unknown" in the PDB file. All assignments are combined into a web-accessible database, which will be updated as soon as the new structures from structural genomics projects are released. Because most of these PDB entries are still not published, we believe that our repository will help to reduce the number of proteins that have functions marked as "unknown" in the PDB file.

### Sequence-function relationship

**Figure 1:**
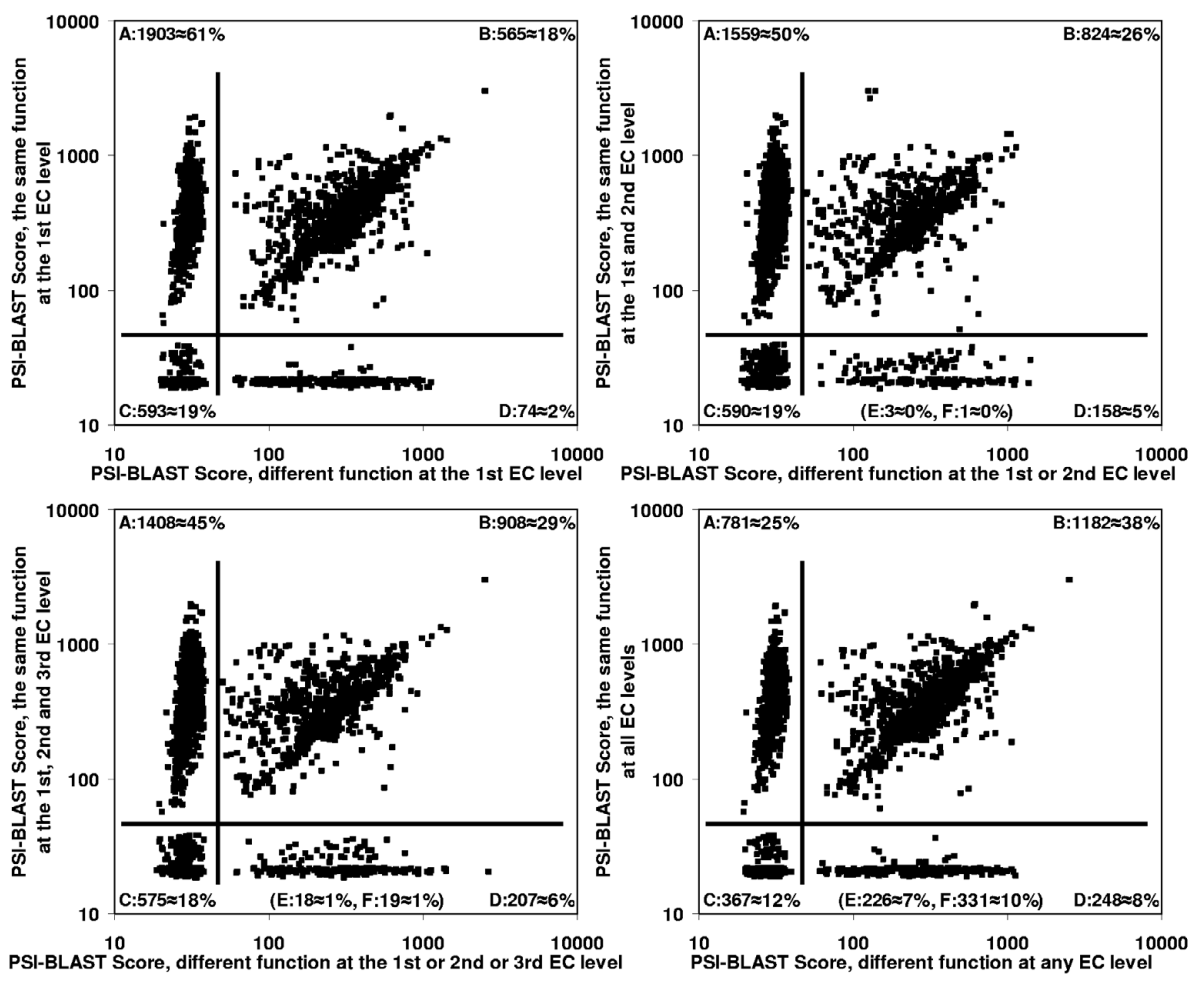
PSI-BLAST score of the most similar protein with **the same **enzyme function **versus **PSI-BLAST score of the most similar protein with **different **enzyme function at the 1^st ^(upper left chart), 2^nd ^(upper right chart), 3^rd ^(lower left chart), and 4^th ^EC level (lower right chart). Calculation was conducted for non-redundant set of 3,135 chain sequences (amino acid identity < 90%) of known structure and enzyme function. Each PSI-BLAST score was taken after the third iteration using 10,278 non-redundant sequence chains (including 3,135) from the Protein Data Bank to build a sequence profile. In each of the charts there are four clusters of points (A, B, C, and D) separated by the horizontal and vertical line. The A and C groups correspond to sequences that are not similar to any enzyme with a different EC number. Two other clusters (B and D) contain proteins from sequence superfamilies that have more than one function. Last two groups (E and F – not shown in the charts) include proteins of orphan function in this dataset. F group contains sequences which are significantly similar to other proteins, while E group corresponds to singleton sequences.

Before predicting the enzyme function based on structural relationship, we checked if it was possible to assign the EC number to the protein using only sequence similarity information. George et al. found that even for homologues detected by a third iteration PSI-BLAST profile there is a 50:50 chance of assigning a fairly specific three-digit EC number [6]. This work seems to be in contrast with many reports suggesting that it is very difficult to successfully infer function below 40% sequence identity [10,11]. Therefore, we conducted an experiment to investigate both claims. Sequence chains from the Protein Data Bank were clustered by similarity using 90% of amino acid identity (AA id.) as a cutoff value. We got 3,135 groups containing one or more proteins with known enzyme function (a total number of clusters >10,000). Next, we calculated a PSI-BLAST alignment score between each of the pairs of the 3,135 representative sequences. 565 (18%) of the proteins were classified to superfamilies that contains at least two enzymes whose EC numbers were different at the first EC level (upper left chart in the Figure 1). But on the other hand, 781 (25%) of the sequences had significant similarity to enzymes with the same EC number at all EC levels, and were not similar to any others (lower right chart in the Figure 1). All the results suggest that there is no general cutoff value of sequence similarity which could be used to assign function to the query. Probably each of the known enzymes should have its own cutoff for function assignments. Here we show that a similar situation is observed when the EC number is predicted using information about structure-function relations.

### Construction and content

#### 3D-Hit algorithm

Two different strategies were applied to annotate the proteins with EC numbers: namely 3D-Hit and 3D-Fun. The first method simply scans using the 3D-Hit program [12] a sequentially non-redundant database of structures that are characterized by four cutoff values. Each value is defined by the highest, known score of structural similarity to any protein with different enzyme function at the corresponding or lower EC level. In the 3D-Hit strategy, the EC number of the protein with the strongest structural similarity is completely (or partially) assigned to the query, if the similarity score is greater than all (or any) of the cutoff values. As an example; let us consider a query protein which has the 3D-Hit score = 150 to the enzyme with the EC number 1.2.3.4 and the cutoff values = 100, 120, 180, 200, respectively. This structure will obtain an EC number assignment of 1.2.?.?.

#### 3D-Fun algorithm

All structural similarity scores are used for annotation in the 3D-Fun strategy. First, the query structure and all sequentially non-redundant proteins are hierarchically clustered (grouped) by structural similarity using complete-link algorithm[13,14]. Next, the EC number is completely (or partially) assigned to each group in each clustering iteration, if all of the enzymes in the group have the same function at all (or any) of the EC levels; otherwise the EC number is assigned as unknown. As an example let us consider a cluster that contains 4 structures: the query protein and 3 enzymes with EC numbers 1.2.3.4, 1.2.3.6, and 1.2.4.1. This cluster will obtain an EC number assignment of 1.2.?.?. For the final prediction, the enzymatic function of the smallest cluster which contains the query structure is used. In the contrary to the 3D-Hit strategy, the 3D-Fun algorithm takes into account the enzymatic function of all structures that have greater values of similarity to the query than to all other proteins of the whole set.

#### Final assignments

We used both presented algorithms to infer the EC number for the 499 proteins from structural genomics that are currently available and have unknown functions. In order to avoid over-annotation due to partial EC numbers we used Green and Karp recommendation [15]. If 3D-Hit and 3D-Fun methods were inconsistent in predicting enzyme function at any EC level it was indicated with a '?' symbol in its corresponding position (e.g. 2.3.4.?). If assignments were fully consistent, we indicated it with an 'n' in the fourth EC level (e.g. 2.3.4.n) which means that exact activity of this enzyme was predicted, but a sequence number has not been yet assigned by the Nomenclature Committee of the International Union of Biochemistry and Molecular Biology (NC-IUBMB).

### Utility and discussion

#### Structure-function relationship

**Figure 2:**
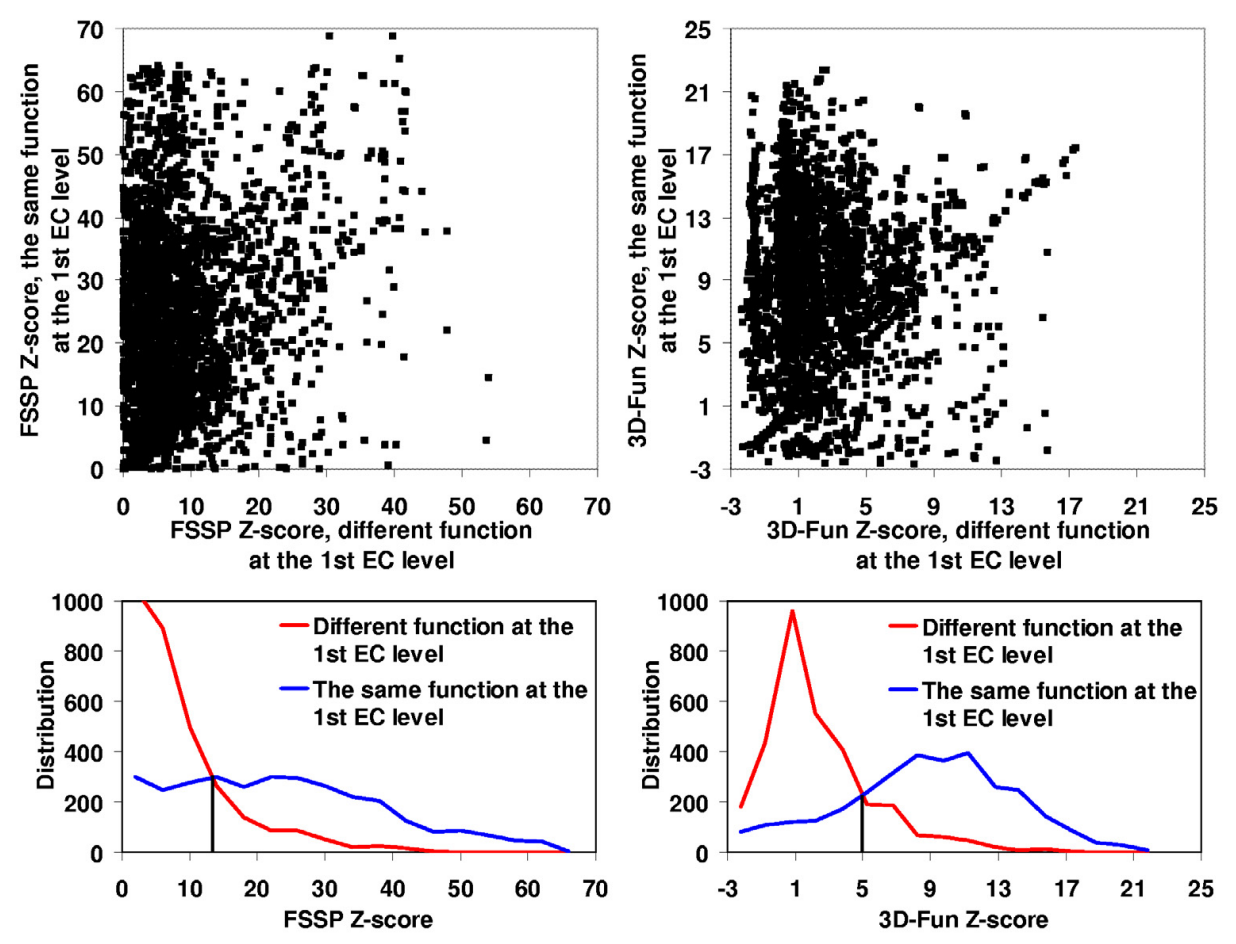
FSSP (on the left) and 3D-Fun (on the right) Z-score of the most similar protein with **the same **enzyme function at the **1^st ^**EC level **versus **Z-score of the most similar protein with **different **enzyme function at the **1^st ^**EC level. Calculation was conducted for 3,135 sequentially non-redundant structures of known function. Corresponding histograms are shown below the charts. If FSSP Z-score = **13 **and 3D-Fun Z-score = **5 **were used as a cutoff value we would obtain sensitivity of **79**% and **83**% with specificity at **74**% and **79**%, respectively.

**Figure 3:**
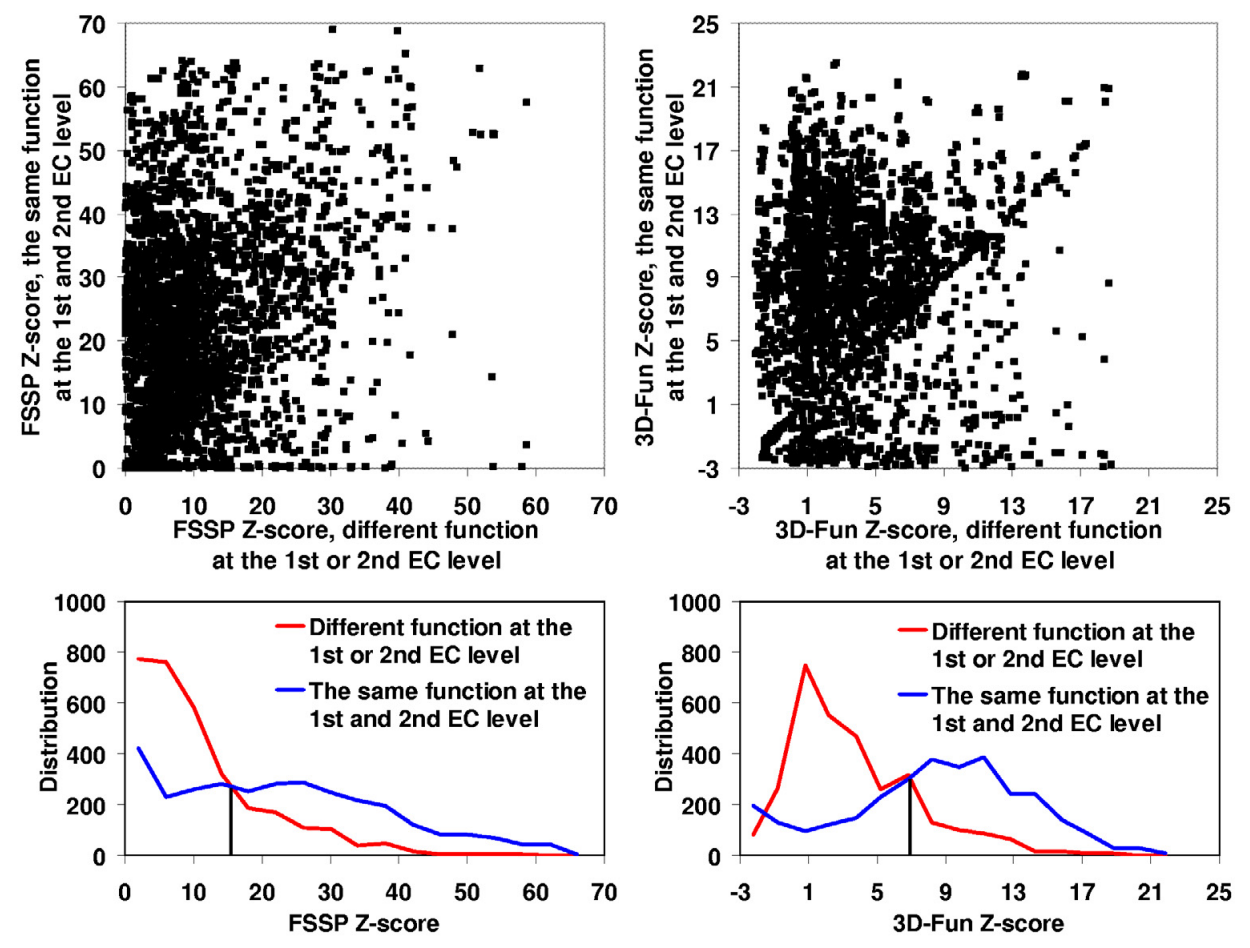
FSSP (on the left) and 3D-Fun (on the right) Z-score of the most similar protein with **the same **enzyme function at the **1^st ^and 2^nd ^**EC level **versus **Z-score of the most similar protein with **different **enzyme function at the **1^st ^or 2^nd ^**EC level. Calculation was conducted for 3,135 sequentially non-redundant structures of known function. Corresponding histograms are shown below the charts. If FSSP Z-score = **15 **and 3D-Fun Z-score = **7 **were used as a cutoff value we would obtain sensitivity of **73**% and **79**% with specificity at **68**% and **70**%, respectively.

**Figure 4:**
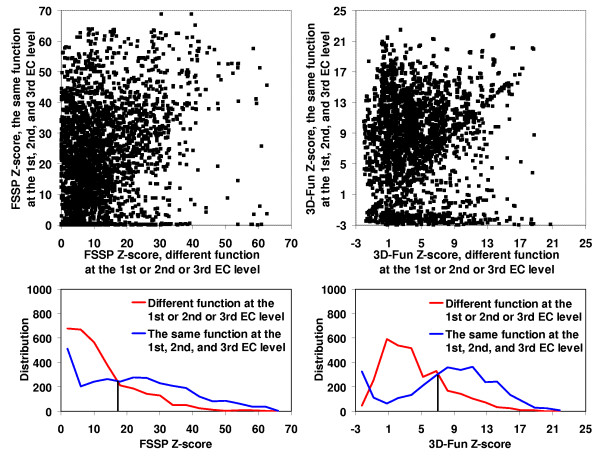
FSSP (on the left) and 3D-Fun (on the right) Z-score of the most similar protein with **the same **enzyme function at the **1^st^**, **2^nd ^and 3^rd ^**EC level **versus **Z-score of the most similar protein with **different **enzyme function at the **1^st ^or 2^nd ^or 3^rd ^**EC level. Calculation was conducted for 3,135 sequentially non-redundant structures of known function. Corresponding histograms are shown below the charts. If FSSP Z-score = **17 **and 3D-Fun Z-score = **8 **were used as a cutoff value we would obtain sensitivity of **70**% and **78**% with specificity at **64**% and **66**%, respectively.

**Figure 5:**
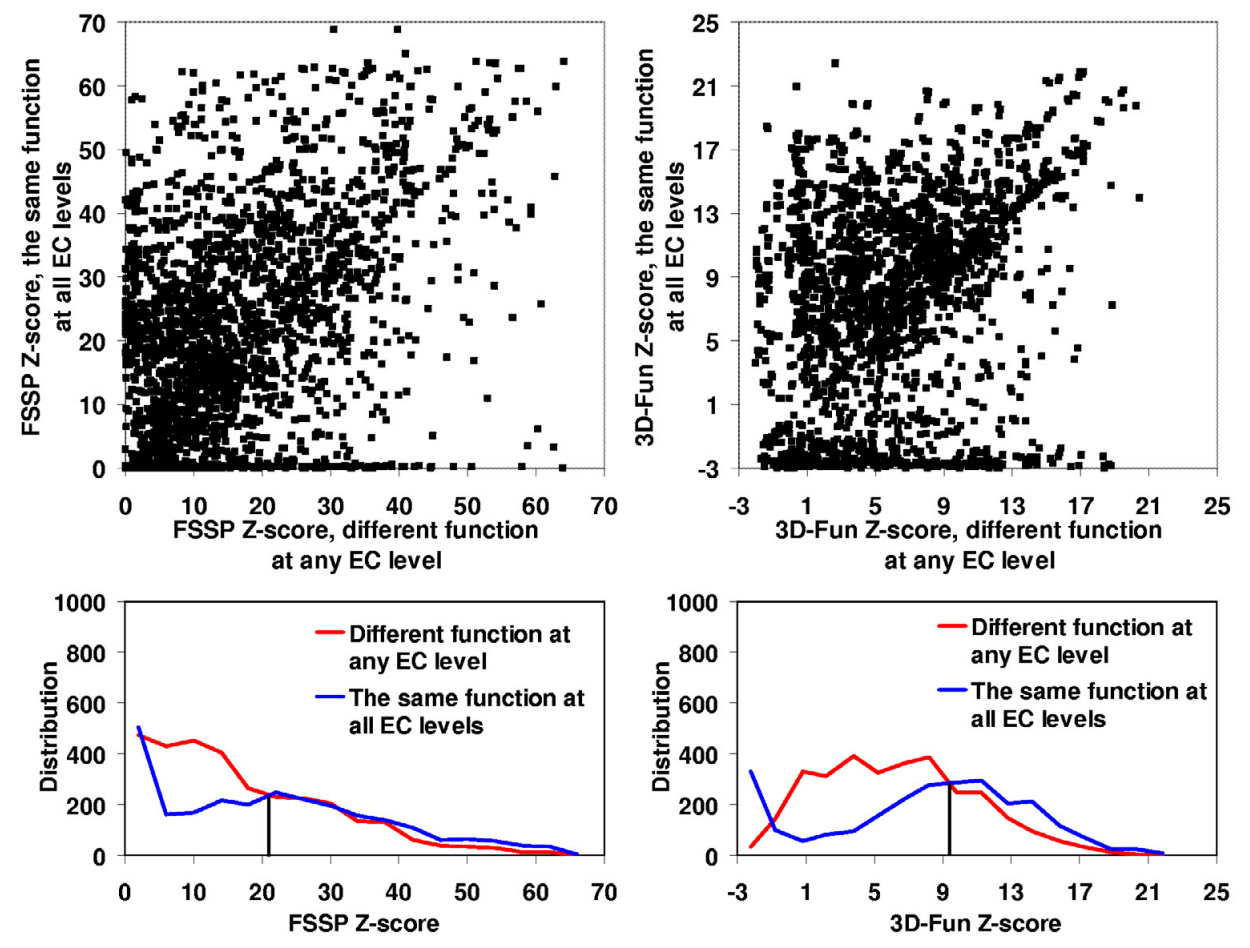
FSSP (on the left) and 3D-Fun (on the right) Z-score of the most similar protein with **the same **enzyme function at the **all **EC level **versus **Z-score of the most similar protein with **different **enzyme function at **any **EC level. Calculation was conducted for 3,135 sequentially non-redundant structures of known function. Corresponding histograms are shown below the charts. If FSSP Z-score = **23 **and 3D-Fun Z-score = **9 **were used as a cutoff value we would obtain sensitivity of **55**% and **60**% with specificity at **53**% and **55**%, respectively.

**Figure 6:**
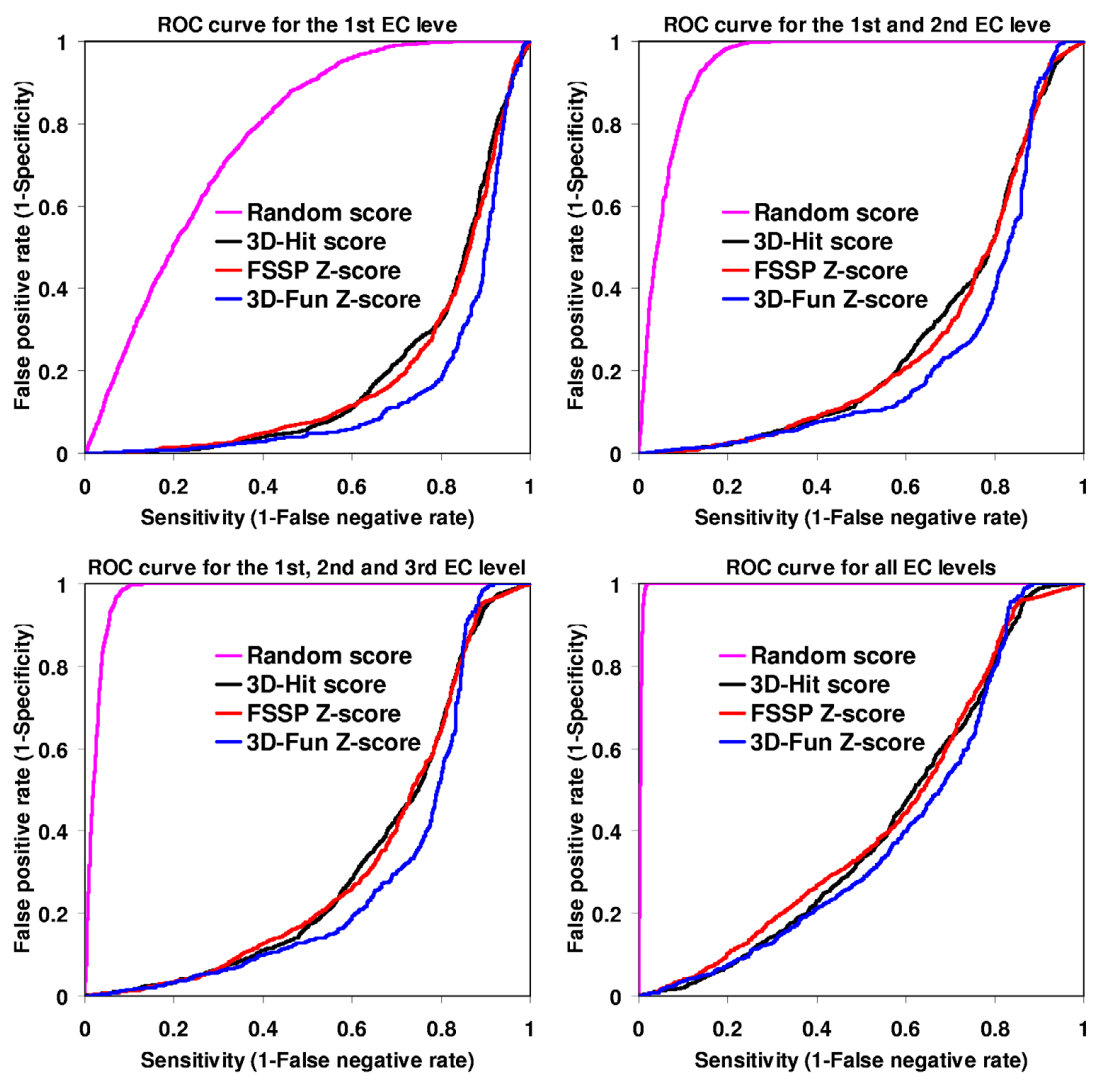
ROC curves for the 1^st ^EC level (upper left chart); 1^st ^and 2^nd ^EC level (upper right chart); 1^st^, 2^nd ^and 3^rd ^EC level (lower left chart) and for all EC levels (lower right chart). Calculation was conducted for 3,135 sequentially non-redundant structures of known function. A random ROC curve (magenta colored) is not a diagonal line (usually presented in ROC plots) because assignment of enzyme function is more complicated than a problem of bimodal classification. Clearly, the probability of assignment of an incorrect EC number is bigger (for the 1^st ^EC level) or much bigger (for all EC levels) than the correct one.

In the Figures 2, 3, 4, 5, we presented a detail comparison of quality of the predicting the EC number based on the 3D-Fun Z-score and the FSSP Z-score [16]. The experiment was performed with 3,135 sequentially non-redundant structures of known enzyme function that were used in the sequence-function test. The Figures show that we would obtain better sensitivity (from 4% to 8%) with better specificity (from 2% to 5%) at all EC levels if we used the 3D-Fun Z-score instead of the FSSP Z-score as a cutoff value for assigning an EC number. This claim is also supported by four ROC curves presented in Figure 6, which portray EC number assignment accuracy of FSSP, 3D-Hit and 3D-Fun algorithms. The most important conclusion of the presented comparisons is the fact that there is not a single, good FSSP or 3D-Fun Z-score cutoff which could be used for assignments of the enzyme functions without human intervention. Of course, we might have used the FSSP Z-score = 50 or the 3D-Fun Z-score = 17 as cutoff and theoretically obtained a false positive rate only slightly greater than zero up to the third EC level. However, to our knowledge, there is no structure of unknown function, which comes from structural genomics, and has similarity to any protein of known structure >50 (FSSP Z-score) or >17 (3D-Fun Z-score). Therefore, we suggest having different cutoffs for different protein folds when similar experiments are carried out. In our case, the cutoff values are defined by the most similar structure with different function.

#### Meta-strategy

**Figure 7:**
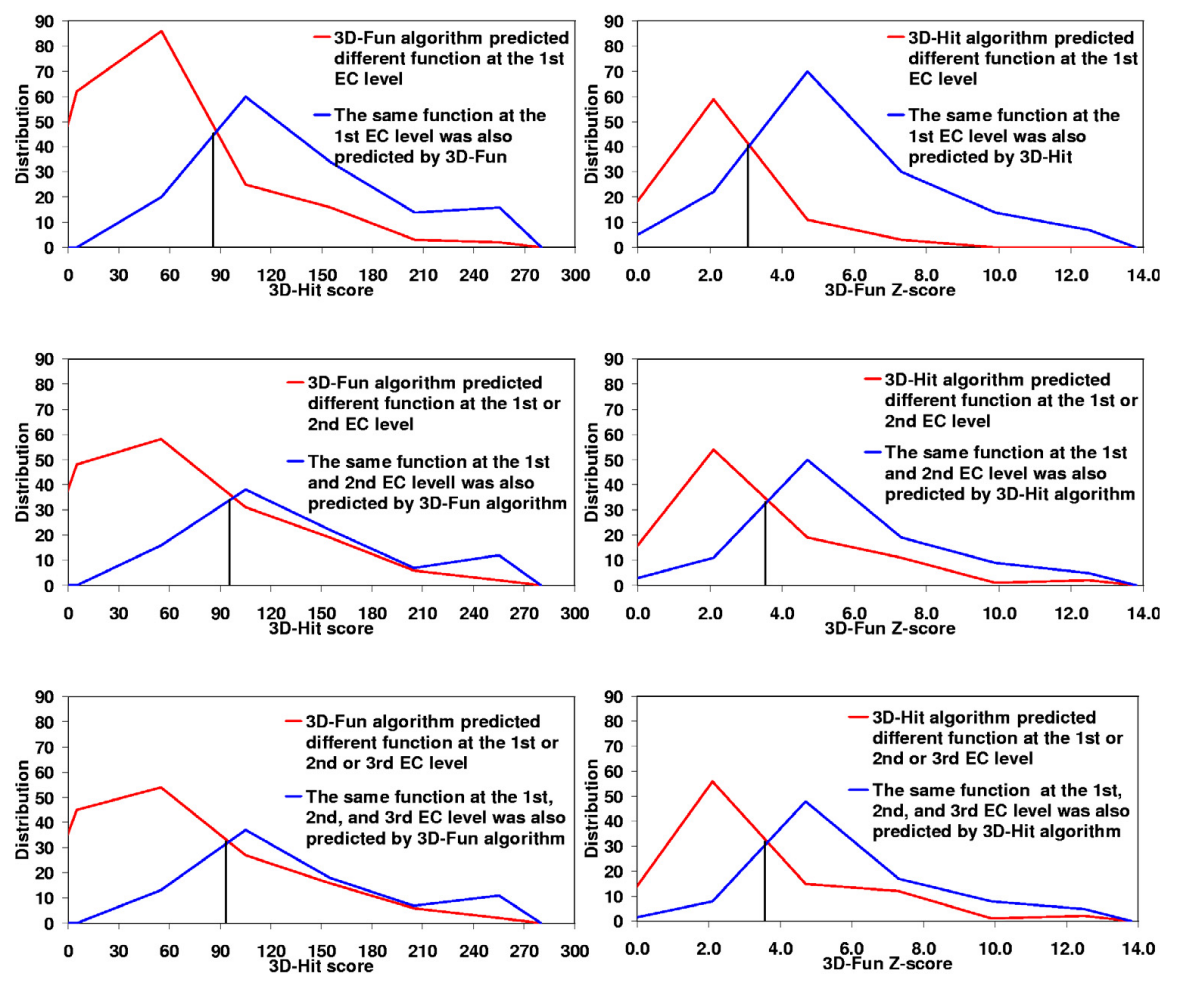
Distribution of consistent (blue lines) and inconsistent (red lines) predictions of an enzyme function conducted using 3D-Hit (left charts) and 3D-Fun (right charts) algorithms. The assignments were performed at the 1^st ^(upper charts), 2^nd ^(middle charts), and 3^rd ^EC level (lower charts) for 499 proteins of unknown function from the structural genomics centers. Marked cutoff values (3D-Hit = 89 and 3D-Fun = 3.1) corresponds to a 50:50 probability of assigning a consistent EC number.

In spite of the fact that the 3D-Hit and 3D-Fun algorithms used fold-specific cutoffs of similarity score, both of them gave conflicting predictions for some of the 499 proteins selected from structural genomics. For example, the EC number was correctly assigned to 1RVK and 1K77 structures only by one program, 3D-Hit or 3D-Fun, respectively. Figure 7 shows distribution of consistent and inconsistent EC number assignments conducted by both methods. This figure justifies the usage of well-known Meta-strategy, which dramatically increased the specificity of sequence similarity search methods in the past [17]. In the set of the 499 structures, we could not find any example of wrong prediction at the first EC level with the 3D-Hit score >89 and the 3D-Fun Z-scores>3.1, which was made incorrectly by both programs in the same way. However one of our reviewers found one example, namely that 1Y7I[18] is now known to be a methyl salicylate esterase (3.1.1.?) while 4.?.?.? (lyase) was predicted by both algorithms.

#### PDB-UF accuracy

Structural genomics initiatives tend to target structures that are less typical of the PDB as a whole and so the cutoffs derived from the whole PDB may not be entirely applicable. Therefore, we analyzed 58 structures with predicted EC numbers, which were recently published and functionally annotated since this may give a truer indication of the accuracy. We found only one additional (except described above) incorrect prediction: 1VGY[19] had been characterized as a succinyl diaminopimelate desuccinylase (3.5.1.?) while metallocarboxypeptidases function (3.4.17.n) was assigned. All such predictions will be manually corrected. However, as more structures are solved in the Protein Data Bank, the PDB-UF method will be more and more accurate and human intervention will not be required.

#### Example of PDB-UF record

**Figure 8:**
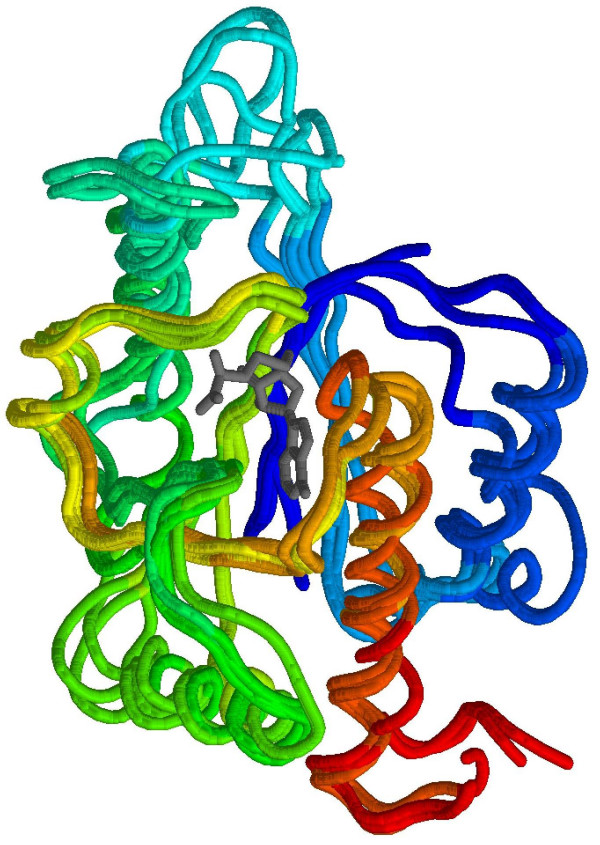
The backbone superposition for 2 deep trefoil knotted TrmD methyltransferases (PDB codes: 1P9P, and 1UAJ) and 4 hypothetical proteins from *A. Aureus*, *E. Coli*, *T. Maritime *and *B. Subtilis *(PDB codes: 1VH0, 1NS5, 1O6D, and 1TO0, respectively). All of the chains are colored from blue (N-termini) to red (C-termini). The *S*-adenosyl-L-homocysteine (AdoHcy) co-factor in 1P9P entry is shown in gray. The highest sequence identity among the group of 4 proteins with unknown function is 54% and the highest identity to the two known methyltransferases is 15%.

Four three-dimensional structures of hypothetical proteins from various species (*A. Aureus*, *E. Coli*, *T. Maritime*, and *B. Subtilis*), which came from different structural genomics consortia, were chosen to demonstrate the utility of the algorithm. The EC numbers of these bacterial proteins have not been assigned in PDBSprotEC and SCOPEC databases. Moreover, standard sequence comparison tools such as PSI-BLAST run against the NCBI non-redundant protein sequence database or RPS-BLAST applied using the Conserved Domain Database[20] failed to assign any function to them. A 3D-Hit structural search detected a strong similarity to a TrmD methyltransferase (MTase) family, represented by the 1P9P[21] and 1UAJ[22] structures. The 3D-Fun program provided similar results by clustering the query model and all TrmD structures into one group with Z-score from 3.64 to 4.22 (depending on the chosen query). Moreover, 3D-Fun found additional similarity to 4 members of a SpoU MTase family. The TrmD and SpoU methyltransferases share a common evolutionary origin and form a single SPOUT (SpoU-TrmD) class[23]. A fold of the SPOUT class is distinct from the consensus MTase fold. All SPOUT proteins contain a deep trefoil knot structure in the catalytic domain and a non-canonical AdoMet/AdoHcy-binding site. A superimposition of 2 TrmD MTases and 4 query structures are presented in Figure 8.

## Conclusion

The PDB-UF database is a collection of assigned EC numbers to protein structures of unknown function, which come from the structural genomics centers. Structure-based prediction of the EC number was conducted having different cutoff values for a different protein folds. In order to reduce the number of false positives the annotation was performed using the Meta-strategy. The web-based repository will be updated automatically when new protein structures are released.

## Availability

The database is available at  and at 

## Authors' contributions

MvG developed the PDB-UF database and the 3D-Fun method. DP and LR designed the 3D-Hit algorithm. KG and ES provided thoughtful insights and helped in testing. All authors have read and approved the manuscript.
